# Characteristics of Seasonal Influenza Virus Activity in a Subtropical City in China, 2013–2019

**DOI:** 10.3390/vaccines8010108

**Published:** 2020-03-01

**Authors:** Aiqin Zhu, Jianhua Liu, Chuchu Ye, Jianxing Yu, Zhibing Peng, Luzhao Feng, Liping Wang, Ying Qin, Yaming Zheng, Zhongjie Li

**Affiliations:** 1 Division of Infectious Disease, Key Laboratory of Surveillance and Early-Warning on Infectious Disease, Chinese Center for Disease Control and Prevention, Beijing 102206, China; zhuaiqin28@163.com (A.Z.); yujianxing@icdc.cn (J.Y.); pengzb@chinacdc.cn (Z.P.); fenglz@chinacdc.cn (L.F.); wanglp@chinacdc.cn (L.W.); qinying@chinacdc.cn (Y.Q.); zhengym@chinacdc.cn (Y.Z.); 2 Yichang Center for Disease Control and Prevention, Yichang 443003, China; amour_1114@163.com; 3 Research Base of Key Laboratory of Surveillance and Early Warning of Infectious Disease, Pudong New Area Center for Disease Control and Prevention, Chinese Center for Disease Control and Prevention, Shanghai 200136, China; cynthia-cloth@163.com

**Keywords:** influenza, subtropical city, seasonality

## Abstract

Background: To optimize seasonal influenza vaccination programs in regions with potentially complicated seasonal patterns, the epidemiological characteristics of seasonal influenza activity in a subtropical city of China were explored. Materials and Methods: Influenza virus data of patients with influenza-like illness (ILI) during 2013–2019 were collected from two sentinel hospitals in a subtropical region of China, Yichang city. The influenza virus positive rate among sampled ILI cases served as a proxy to estimate influenza seasonal characteristics, including periodicity, duration, peaks, and predominant subtypes/lineages. Epidemiological features of different years, seasons and age groups were analyzed, and vaccine mismatches were identified. Results: In total, 8693 ILI cases were included; 1439 (16.6%) were laboratory-confirmed influenza cases. The influenza A positive rate (10.6%) was higher than the influenza B positive rate (5.9%). There were three influenza circulation patterns in Yichang: (1) annual periodicity (in 2013–2014, 2015–2016 and 2018–2019), (2) semiannual periodicity (in 2014–2015), and (3) year-round periodicity (in 2016–2017 and 2017–2018). Summer epidemics existed in two of the six years and were dominated by influenza A/H3N2. Winter and spring epidemics occurred in five of the six years, and A/H1N1, A/H3N2, B/Victoria, and B/Yamagata were codominant. During the study period, the predominant lineages, B/Victoria in 2015-16 and B/Yamagata in 2017–2018, were both mismatched with the influenza B component of the trivalent vaccine. Children 5–14 years old (26.4%) and individuals over 60 years old (16.9%) had the highest influenza positive rates. Conclusions: The seasonal epidemic period and the predominant subtype/lineage of influenza viruses in Yichang city are complex. Influenza vaccination timing and strategies need to be optimized according to the local features of influenza virus activity.

## 1. Introduction

Influenza is one of the most serious diseases worldwide, resulting in an estimated 1 billion cases, 3-5 million hospitalizations, and 290,000–650,000 respiratory deaths globally per year [1]. The influenza vaccine is the most effective way to prevent influenza and increasing coverage could significantly reduce influenza-associated morbidity and mortality [2,3]. Trivalent and quadrivalent influenza vaccines are available in China, though they are not currently included in China’s National Expanded Immunization Program, and citizens in most cities of China must pay an out-of-pocket fee for these vaccinations [2,4,5]. Vaccine coverage is still relatively low, at approximately 2% in China, which has a single annual influenza vaccination campaign, and the technical guidelines for seasonal influenza vaccination recommend individuals to get vaccinated by the end of October [2,5,6]. Understanding the seasonality of influenza is important for policy making and guiding control strategies because it determines the timing of the influenza vaccination campaign before the influenza season and allows vaccine recipients to develop a protective immune response [7].

China has a vast area, complex climatic conditions, and a large population, and the seasonality of influenza activity varies from region to region. There are three climatic zones in China: Temperate, subtropical, and tropical. Previous studies have shown that the seasonality of influenza in subtropical and tropical zones is more variable than that in temperate zones [3,8,9,10]. In temperate regions, influenza epidemics occur annually and with predictable seasonality; in tropical regions, they can occur year round, with unpredictable peaks [11,12]. In the global influenza surveillance system, China is the only country with different seasonal zones that systematically conducts influenza surveillance. Some recent studies have discussed influenza seasonality in subtropical regions; however, most of them concentrated on large cites [13,14,15]. Influenza seasonality and periodicity can vary by location, population at risk, time period, and epidemic features in subtropical regions, where relatively small population sizes and low population movement are highly complex and relatively unknown [15,16,17,18]. This study aims to understand influenza seasonality in a subtropical city of China and to provide further evidence for optimizing influenza vaccination programs at the subnational level.

## 2. Materials and Methods

### 2.1. Influenza Virus Surveillance

From 2005 to 2019, year-round influenza-like illness (ILI) surveillance was conducted in Yichang, a central city with a subtropical climate and a population of 3.9 million in 2017 located in Hubei Province (110°-112°E, 29°-31°N) [19]. ILI surveillance data were collected from two sentinel hospitals, one is Central Hospital, the other one is Second People’s Hospital, which has been part of the network since 2017. All data was provided by Yichang Center for Disease Control and Prevention(Yichang CDC). ILI was defined as a measured temperature ≥38 °C with the presence of either cough or sore throat. Every week, the medical staff working in the internal medicine clinics, internal medicine emergency clinics, fever clinics, pediatric internal medicine clinics and pediatric emergency clinics of the two sentinel hospitals screened patients to identify those meeting the case definition.

In each sentinel hospital, nasopharyngeal swabs were collected on Tuesdays and Thursdays from the first 5 or 20 ILI cases for influenza virus testing, resulting in an average of 10 to 40 specimens per hospital per surveillance week.

Samples were stored at 4 °C to 8 °C and sent to the laboratory at the Yichang CDC for testing within 24 h. Reverse transcription PCR (RT-PCR) assays were performed to identify the types/subtypes of influenza virus, following a standard protocols [20]. B lineage identification was not performed in all the samples during the first three years.

### 2.2. Data Analysis

We analyzed data from sampled ILI patients from June 3, 2013, to June 2, 2019; the collected data included sex, age, date of illness onset and laboratory testing results. The positive rate was calculated by dividing the number of samples positive for influenza virus by the total number of samples tested. The age-specific influenza positive rate by subtype/lineage was calculated as the number of sentinel specimens testing positive for each subtype/lineage (numerator) among the ILI cases recruited for specimen collection (denominator) in each corresponding age group [21].

A surveillance year was defined as the period ranging from calendar week 23 of one year (approximately the week of June 1) to calendar week 22 of the next year (approximately the week of May 31). The surveillance week started on Monday and ended on Sunday. According to the seasonal climate in Yichang, the division of seasons was accurate to the week. The 12th week to the 22nd week of each year is spring, the 23rd week to 38th week is summer, the 39th week to the 48th week is autumn, and the 49th week to 11th week of the following year is winter [22].

There are many different analytic approaches to assess influenza seasonality, and a universal definition and methodology is lacking. Our study used a threshold to define influenza epidemic and nonepidemic periods because it was easy to understand and compare with other studies [7,12,15,23]. The start of the influenza epidemic period was defined as the first week during which the positive rate of influenza virus testing was higher than 10% and remained above that level for at least four consecutive weeks, and the end of an influenza epidemic period was defined as the first week during which the positive rate was lower than 10% and remained at that level for at least four consecutive weeks [7,12,13]. The duration of the influenza epidemic was defined as the number of weeks between the start and end of the influenza epidemic period. The occurrence of only one epidemic period per surveillance year was defined as annual periodicity. Two epidemic periods in a surveillance year was classified as semiannual periodicity, and an epidemic period lasting more than two seasons was defined as year-round periodicity. The week with the highest positive rate of influenza virus was identified as the peak of the epidemic period. The predominant subtype/lineage was defined as the subtype/lineage with the highest positive rate in two consecutive weeks. To determine whether the predominant lineage matched the recommended lineage, the influenza B lineage virus component in the trivalent influenza vaccine recommended for the Northern Hemisphere was compared to the predominant influenza virus B lineage circulating in the study area.

Data were analyzed using Stata SE 12 (StataCorp, College Station, TX). The chi-square test or Fisher’s exact test was used for categorical variables as appropriate. A p-value of <0.05 was considered statistically significant.

## 3. Results

### 3.1. Overall Characteristics of Influenza Virus Surveillance

From 2013–2019, 8693 ILI patients were enrolled for influenza virus surveillance in this study. Of them, 3836 (44.1%) were males and 4857 (55.9%) were females. The median age was 22 years (interquartile range (IQR): 6–36 years old). The overall positive rate of influenza virus was 16.6%; the highest positive rate (26.0%) occurred in the 2017–2018 surveillance year, and the lowest rate (9.1%) occurred in the 2013–2014 year. The positive rate of influenza A was 10.6%, among which the positive rate of A/H1N1 was 5.0% and that of A/H3N2 was 5.6%. The positive rate of influenza B was 5.9%, among which the positive rate of B/Yamagata was 2.9% and that of B/Victoria was 1.9% (Table 1 and Supplementary Material Table S1).

### 3.2. Influenza Virus Activity among Age Groups

During the six study years, the influenza positive rate varied by age group (*p* < 0.001), and the overall influenza positive rate was highest among children 5–14 years old (26.4%), followed by elderly adults ≥60 years old (16.9%), those 25–59 years old (15.9%), those 15–24 years old (13.1%), and children younger than 0–4 years old (12.1%) (Table 2 and Supplementary Material Table S2 and S3).

For influenza A virus, the positive rate of A/H3N2 (7.6%) was much higher than that of A/H1N1 (4.0%) among elderly people over 60 years old. For influenza B virus, the positive rate of B/Victoria peaked in patients 5–9 years old (4.4%) and 30~34 years old (3.3%), and B/Victoria was rarely detected among people ≥40 years old (0.3%). However, B/Yamagata peaked in children 10–14 years old (6.3%) and then decreased until it re-increased in those 30 years old and finally peaked among 70–74 year-olds (5.9%) (Table 2 and Supplementary Material Table S2 and S3).

### 3.3. Seasonality Patterns of Influenza Viruses

**Figure 1:**
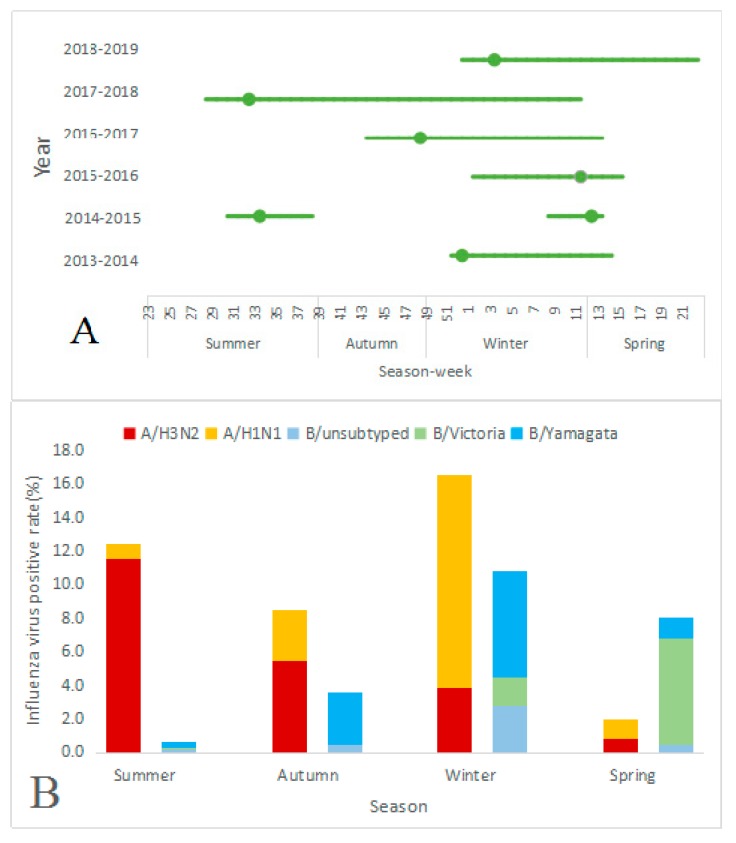

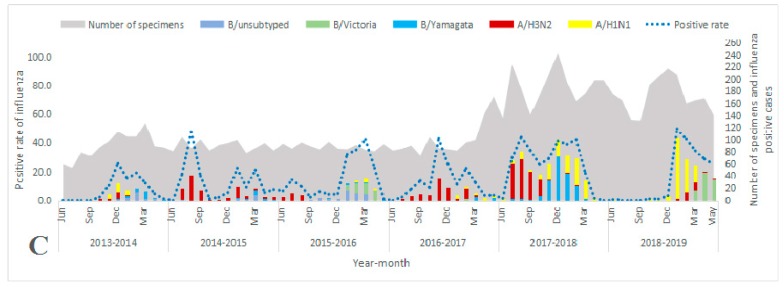
(**A**) Influenza epidemic duration and peak times in Yichang, 2013–2019, using the positive rate as a proxy. The dark blue dot indicates the peak time; (**B**) Distribution of positive rates by subtypes/lineages of influenza viruses A and B in different seasons; (**C**) Temporal trends of influenza virus activity by subtype/lineage. The shaded area represents the total number of specimens tested. The dark blue dotted line indicates the positive rate of influenza.

During the six-year surveillance period, three epidemic periodicities of seasonal influenza viruses were identified: (1) Three years had annual periodicity (2013–2014, 2015–2016, and 2018–2019); (2) one year had semiannual periodicity (2014–2015); and (3) the remaining two years had year-round periodicity (2016–2017 and 2017–2018) (Table 3 and Figure 1).

Annual periodicity occurred in winter and spring; the median start time was week 52, the median end time was week 16, and the median duration was 16 weeks. In all three years, the estimates of influenza peaks were in winter, with a median peak in week 4. In the surveillance years 2013–2014, 2015–2016, and 2018–2019, the influenza positive rates at the peak times were 45.8% (11/24), 54.6% (12/22), and 72.5% (29/40), respectively (Table 3 and Figure 1).

Semiannual periodicity was identified in surveillance year 2014-15; one epidemic was identified in summer, and the other epidemic was identified in winter to spring. The influenza epidemic in the summer season peaked at week 33, and the positive rate in that week was 55.6% (15/27). The peak in the winter and spring seasons occurred in week 12, in which the positive rate was 31.8% (7/22). The epidemic durations of those with semiannual periodicity were shorter than those with annual periodicity; epidemics lasted for 9 weeks in the summer season and 6 weeks in the winter and spring seasons versus 16 weeks with annual periodicity. The epidemic in winter and spring started in week 8, which was also later than that the start of epidemics with annual periodicity (Table 3 and Figure 1).

Regarding year-round periodicity in two consecutive surveillance years, 2016-17 and 2017-18, the influenza epidemic started in different seasons. In 2016–2017, the epidemic started in autumn, while in 2017–2018, the epidemic started in summer. However, the end times were similar, at week 14 in 2016–2017 and week 12 in 2017–2018. Further, the duration of the influenza epidemic in 2016–2017 was shorter than that in 2017–2018 (23 weeks and 36 weeks, respectively). The peaks occurred in the early phases of the epidemic period, which were autumn and summer in surveillance years 2016–2017 and 2017–2018, respectively, and the positive rates at the peak times were 54.6% (12/22) and 59.2% (29/49), respectively (Table 3 and Figure 1).

### 3.4. Characteristics of Seasonal Influenza Epidemics by Season

The positive rate of influenza differed significantly by season (*p* < 0.001). The highest influenza positive rate occurred in winter (27.3%, 729/2666), followed by summer (13.0%, 314/2412) and autumn (12.0%, 207/1727), and it was lowest in spring (10.0%, 189/1888). In summer, autumn and winter, the positive rates of influenza A were higher than those of influenza B, while in spring, the opposite was true. The positive rate of influenza A in summer was 12.4% (299/2412); the positive rate of A/H3N2 was 11.5% (278/2412), which was nearly twelve times higher than the rate of A/H1N1 (0.9%, 21/2412). The positive rate of influenza B in summer was 0.6% (15/2412), which was one-twentieth the rate of influenza A. In autumn, the positive rates of influenza A and influenza B were 8.5% (146/1727) and 3.5% (61/1727), respectively, among which the positive rate of A/H3N2 was 5.4% (94/1727), A/H1N1 was 3.0% (52/1727), B/Yamagata was 3.1% (53/1727) and B lineage identification not performed was 0.5% (8/1727). In winter, the positive rate of influenza A was 16.5% (440/2666). In contrast with summer and autumn, the positive rate of A/H1N1 (12.6%, 337/2666) in winter was higher than that of A/H3N2 (3.9%, 103/2666). The positive rate of influenza B was 10.8% (289/2666), and the rate of B/Yamagata (6.4%, 170/2666) was higher than those of B/Victoria (1.7%, 45/2666) and B lineage identification not performed (2.8%, 74/2666). In spring, the positive rate of influenza A was 2.0% (37/1888) and of influenza B was 8.1% (152/1888); the rate of B/Victoria was four times higher than that of B/Yamagata (6.4% versus 1.2%) (Table 3 and Figure 1).

### 3.5. Matching with the Trivalent Influenza Vaccine

The predominant influenza virus B lineage was mismatched with the influenza B virus component in the trivalent influenza vaccine recommended for the Northern Hemisphere in two of the six surveillance periods; however, in one of the six years, influenza B was not the predominant lineage. In 2015–2016, the predominant lineage in the Yichang influenza epidemic period was B/Victoria, and the recommended trivalent vaccine contained B/Yamagata; this result was opposite in the 2017–2018 surveillance year (Table 3).

## 4. Discussion

Our findings demonstrate that Yichang may have three different influenza circulation patterns; annual, semiannual and year-round epidemics all occurred in the surveillance years 2013–2019. Summer peaks were mostly attributed to influenza A, mainly A/H3N2, and influenza B was more prevalent in spring. In two of the six years, the influenza B lineage was mismatched with the influenza B virus components in the trivalent vaccines. The highest positive rate of influenza was found in children aged 5–14 years and elderly individuals older than 60 years.

During the study period, the highest influenza positive rate in the tested ILI patients was found in school-aged children who were 5–14 years old (26.4%), followed by those older than 60 years of age (16.9%); the lowest rate was found in children aged less than 5 years (12.1%). A relatively high B/Yamagata percentage was found in older adults in the present study. These results are consistent with the results of recent studies in China, and which may be explained by exposure to the virus early in life and genetic difference [13,14,21]. In China, influenza vaccination is mostly self-paid, with all-age coverage as low as 2% [5]. To decrease the risk of severe infections and complications due to influenza virus, free vaccination policies for elderly individuals have been adopted in some areas, but few policies have been adopted for school-aged children [4,24]. Yichang does not have influenza vaccination policies for elderly individuals or children. Considering the high positive rate of influenza among school-age children, it might be beneficial to prioritize influenza vaccination for school-aged children and implement school-based interventions to reduce the burden of influenza [25,26].

Yichang seems to have a more complex and irregular circulation pattern than other cities in China or subtropical cities [13,15,27], with annual, semiannual and year-round epidemics. A previous study indicated that the midlatitude area of mainland China had unclear seasonal characteristics, which were more complicated than those in the northern (latitude >33° N) and southern (latitudes <27° N) areas in China [15,28]. However, the characteristics of influenza activity in different seasons are relatively clear. Summer was predominated by influenza A, and influenza B was rarely detected; in autumn and winter, influenza A and B were both dominant, and in spring, influenza B dominated. It seemed that influenza B occurred later than influenza A, which was also found in other studies in the Northern Hemisphere [7,13,14,29]. We also found that during the six-year study period, influenza B did not match the recommended strain in two of the years. The World Health Organization makes biannual recommendations for the composition of seasonal influenza vaccines for the Northern Hemisphere and Southern Hemisphere [30]. Until recently, the trivalent vaccine included influenza A/H1N1, A/H3N2, and B strains, with only one of the two B lineages represented. However, a quadrivalent vaccine comprising two influenza A and B lineages was licensed in China in 2018–2019 [2]. In a global study, during seasons in which influenza B was dominant or cocirculated, a vaccine mismatch was observed in 25% of the seasons [31].

As mentioned previously, vaccination is the most effective way to prevent influenza virus infection. Currently, the vaccination coverage rate in Yichang is similar to that in mainland China, which is as low as 2%, and it is recommended that people receive influenza vaccination by the end of October. Even if not vaccinated in October, target populations, especially high-risk groups, should continue to be advocated for vaccination [2]. However, the vaccination time of influenza in Yichang is from October to February of the following year. Given that the antibody titer begins to decline at 6 to 8 months after vaccination and the possibility of year-round circulation, the timing of influenza programs in Yichang may need to be evaluated and making vaccination available beyond February according to the seasonality pattern may be necessary. Considering the potential for vaccine mismatch, encouraging influenza vaccine administration and the availability of a quadrivalent vaccine is beneficial for expanding options for influenza prevention and control.

China is a climatologically and economically diverse country that contains three climatic zones. The current single annual influenza vaccination campaign that occurs at the same time across the whole country may not promote maximum vaccine efficacy, especially in subtropical areas with complex influenza seasonality patterns. Our study suggests that region-specific influenza vaccination programs are needed and provides evidence for optimizing vaccination strategies at the subnational level.

Our results are subject to the following limitations. First, we chose the positive rate to represent influenza activity level in our study. However, we do not know if the positive rate in persons tested is representative of the positive rate in persons not tested, and this index might be influenced by the sampling method and healthcare-seeking behavior. Moreover, the ILI definition was used for sampling, but for some subpopulations, the definition of ILI was not sensitive; for example, the fever symptoms of ILI in elderly individuals may be atypical and may not actually reflect the true level of activity of influenza in different subpopulations [32,33,34,35,36]. Second, influenza B lineage identification, which differentiated B Yamagata and B Victoria, was only performed in some samples during the first three years; the remainder of the samples were identified as only influenza B. Thus, we do not know if the lineage pattern of the identified samples was the same as that in the nonidentified samples. However, considering its distribution, we suppose that this had little influence on our conclusion. Another limitation is that we do not know the vaccination status of the population, so we are unable to comment on differences in influenza positive rates among those who were vaccinated versus unvaccinated. Nevertheless, our study based on PCR-confirmed tests and continuous sampling of all age groups clearly showed that the circulation of both influenza A and B types varied by age group and season in a subtropical region of China. Despite these limitations, few studies provide a comprehensive analysis of both influenza type and subtype seasonality patterns in the midlatitude area of mainland China.

## 5. Conclusions

In summary, our findings demonstrated that the positive rate of influenza varied by age group and season. Yichang may have multiple influenza circulation patterns, and the influenza vaccination program in Yichang could be optimized by adjusting vaccination times and prioritizing school-age children and the elderly population.

## Figures and Tables

**Table 1:** Demographic characteristics of influenza-like illness (ILI) ^1^ cases from two sentinel surveillance sites in Yichang, Hubei, 2013-2019.

Characteristics	2013–2014	2014–2015	2015–2016	2016–2017	2017–2018	2018–2019	Overall
No. of patients	1071	1077	1076	1187	2239	2043	8693
Sex**, *n* (%)**							
M	444 (41.5)	465 (43.2)	463 (43.0)	525 (44.2)	1016 (45.4)	923 (45.2)	3836 (44.1)
F	627 (58.5)	612 (56.8)	613 (57.0)	662 (55.8)	1223 (54.6)	1120 (54.8)	4857 (55.9)
Age, y, median (IQR)	25 (8, 40)	19 (5, 35)	20 (5, 34)	22 (5, 37)	21 (7, 36)	23 (7, 34)	22 (6, 36)
Age group (years), *n* (%) ^❉^							
0–4	161 (15.0)	254 (23.6)	233 (21.7)	277 (23.3)	401 (17.9)	364 (17.8)	1690 (19.4)
5–14	167 (15.6)	203 (18.9)	213 (19.8)	211 (17.8)	404 (18.0)	315 (15.4)	1513 (17.4)
15–24	192 (17.9)	162 (15.0)	184 (17.1)	162 (13.7)	440 (19.7)	404 (19.8)	1544 (17.8)
25–59	478 (44.6)	388 (36.0)	384 (35.7)	449 (37.8)	824 (36.8)	842 (41.2)	3365 (38.7)
60+	73 (6.8)	70 (6.5)	62 (5.8)	88 (7.4)	170 (7.6)	118 (5.8)	581 (6.7)
Weekly number of positive cases, median (IQR)	4 (1, 6)	2 (1, 5.5)	3 (1, 7)	3 (1, 5)	13 (7, 21)	11 (6, 15)	4 (2, 10)
Weekly number of samples tested, median(IQR)	20.5 (16, 24)	20.5 (17, 24.5)	21 (18, 24)	21.5 (17, 26.5)	43.5 (37, 48.5)	38 (32.5, 42.5)	24 (19, 36)

^1^ ILI = influenza-like illness; IQR = interquartile range, ^❉^ = Some columns do not add up to 100% because of rounding.

**Table 2:** Age-specific influenza positive rates in different surveillance years, Yichang, Hubei, 2013–2019.

Age group (years) ^❉^, *n*1 *, *n*2 ^#^ (%)	2013–2014	2014–2015	2015–2016	2016–2017	2017–2018	2018–2019	Overall
0–4	161, 12 (7.5)	254, 28 (11.0)	233, 31 (13.3)	277, 18 (6.5)	401, 82 (20.4)	364, 33 (9.1)	1690, 204 (12.1)
5–14	167, 47 (28.1)	203, 49 (24.1)	213, 56 (26.3)	211, 40 (19.0)	404, 137 (33.9)	315, 71 (22.5)	1513, 400 (26.4)
15–24	192, 8 (4.2)	162, 11 (6.8)	184, 20 (10.9)	162, 20 (12.3)	440, 90 (20.5)	404, 53 (13.1)	1544, 202 (13.1)
25–59	478, 25 (5.2)	388, 45 (11.6)	384, 45 (11.7)	449, 50 (11.1)	824, 213 (25.8)	842, 157 (18.6)	3365, 535 (15.9)
60+	73, 5 (6.8)	70, 10 (14.3)	62, 3 (4.8)	88, 11 (12.5)	170, 60 (35.3)	118, 9 (7.6)	581, 98 (16.9)
Total	1071, 97 (9.1)	1077, 143 (13.3)	1076, 155 (14.4)	1187, 139 (11.7)	2239, 582 (26.0)	2043, 323 (15.8)	8693, 1439 (16.6)

* = Number of ILI patients tested; ^#^ = Number of influenza-positive cases; ^❉^ = Some columns do not add up to 100% because of rounding.

**Table 3:** Characteristics of seasonal influenza virus epidemics and matching with the trivalent influenza vaccine in Yichang, Hubei, 2013–2019.

Characteristics	2013–2014	2014–2015		2015–2016	2016–2017	2017–2018	2018–2019
Epidemic periodicity	Annual	Semiannual		Annual	Year-round	Year-round	Annual
Epidemic period	2013.12.16– 2014.4.7	2014.7.21– 2014.9.22	2015.2.16– 2015.3.30	2016.1.4– 2016.4.18	2016.10.24– 2017.4.3	2017.7.10– 2018.3.19	2018.12.24– 2019.6.2
Epidemic season	Winter, Spring	Summer	Winter, Spring	Winter, Spring	Autumn, Winter Spring	Summer, Autumn Winter	Winter, Spring
Start time (week number)	51	30	8	1	43	28	52
End time (week number)	15	39	14	16	14	12	22
Duration * (number of weeks)	16	9	6	15	23	36	23
Peak time (week number)	52	33	12	11	48	32	4
Predominant subtypes/ lineages	A/H1N1, B/Yamagata	A/H3N2	B/Yamagata	B/Victoria	A/H3N2	A/H3N2, B/Yamagata, A/H1N1	A/H1N1, B/Victoria
Recommended B lineage component in trivalent influenza vaccine	B/Yamagata	B/Yamagata		B/Yamagata	B/Victoria	B/Victoria	B/Victoria
Matching ^#^	Yes	Yes		No	-	No	Yes

* = Duration does not contain the time of the end week (except 2018–2019, as the epidemic was ongoing at the end of the study); ^#^ = The matching results of the influenza B lineage virus component in the trivalent influenza vaccine recommended for the Northern Hemisphere with the predominant influenza virus B lineage circulating in Yichang. “-” in the matching results indicated influenza B was not the predominant lineage in the year 2016-2017.

## Supplementary Materials

The following are available online at https://www.mdpi.com/2076-393X/8/1/108/s1, Figure S1: Location of Yichang city, Hubei Province, China. Figure S2: Temporal trends of influenza virus activity by type, Yichang, Hubei, 2013–2019. Influenza virus-positive cases by virus type (influenza viruses A and B). The shaded area represents the total number of specimens tested. The dark blue dotted line indicates the positive rate of influenza. Figure S3: Distribution of positive rates by subtypes/lineages of influenza viruses A and B in different seasons in the six-year study period. Figure S4: Heat map of influenza virus activity in Yichang, Hubei, 2013–2019. Table S1: Characteristics of seasonal influenza virus in Yichang, Hubei, 2013–2019. Table S2: Age-specific influenza positive rate, Yichang, Hubei, 2013–2019. Table S3: Detailed results of positive rates by age group and subtype/lineage, Yichang, Hubei, 2013-2019. Table S4: Age distribution of influenza-positive patients in different surveillance years, Yichang, Hubei, 2013–2019.
