# Supplementary material for: Characteristics of Seasonal Influenza Virus Activity in a Subtropical City in China, 2013–2019
Source: Vaccines (Basel). 2020 Mar 1;8(1):108. doi: 10.3390/vaccines8010108 (PMC7157579; doi:10.3390/vaccines8010108)
Supplement: Supplementary file 1 [file vaccines-08-00108-s001.pdf]

vaccines

MDPI

Supplemental Materials

Table S1. Characteristics of seasonal influenza virus in Yichang, Hubei, 2013-2019.

Characteristics	2013-2014	2014-2015	2015-2016	2016-2017	2017-2018	2018-2019	Overall
No. of patients	1071	1077	1076	1187	2239	2043	8693
**Type/subtype/lineage**※							
Influenza virus, n（%）	97(9.1)	143(13.3)	155(14.4)	139(11.7)	582(26.0)	323(15.8)	1439(16.6)
Influenza A, n（%）	45(4.2)	109(10.1)	34(3.2)	120(10.1)	388(17.3)	226(11.1)	922(10.6)
A/H1N1	29(2.7)	0(0)	8(0.7)	14(1.2)	189(8.4)	192(9.4)	432(5.0)
A/H3N2	16(1.5)	109(10.1)	26(2.4)	106(8.9)	199(8.9)	34(1.7)	490(5.6)
Influenza B, n（%）	52(4.9)	34(3.2)	121(11.2)	19(1.6)	194(8.7)	97(4.7)	517(5.9)
B/Victoria	0(0)	0(0)	61(5.7)	7(0.6)	3(0.1)	97(4.8)	168(1.9)
B/Yamagata	26(2.4)	18(1.7)	8(0.7)	12(1.0)	191(8.5)	0(0)	255(2.9)
B lineage not detected	26(2.4)	16(1.5)	52(4.8)	0(0)	0(0)	0(0)	94(1.1)

※, Some columns do not add up to 100% because of rounding.

Table S2. Age-specific influenza positive rates, Yichang, Hubei, 2013-2019.

Age group	0-4	5-14	15-24	25-59	60+	Overall
No. of patients	1690(19.4)	1513(17.4)	1544(17.8)	3365(38.7)	581(6.7)	8693
Type/subtype/lineage*						
Influenza virus, n（%）	204(12.1)	400(26.4)	202(13.1)	535(15.9)	98(16.9)	1439(16.6)
Influenza A, n（%）	140(8.3)	221(14.6)	126(8.2)	368(10.9)	67(11.5)	922(10.6)
A/H1N1	55(3.3)	110(7.3)	57(3.7)	187(5.6)	23(4.0)	432(5.0)
A/H3N2	85(5.0)	111(7.3)	69(4.5)	181(5.4)	44(7.6)	490(5.6)
Influenza B, n（%）	64(3.8)	179(11.8)	76(4.9)	167(5.0)	31(5.3)	517(5.9)
B/Victoria	20(1.2)	61(4.0)	21(1.4)	65(1.9)	1(0.2)	168(1.9)
B/Yamagata	29(1.7)	84(5.6)	46(3)	69(2.1)	27(4.7)	255(2.9)
B lineage not detected	15(0.9)	34(2.3)	9(0.6)	33(1)	3(0.5)	94(1.1)

*, Some columns do not add up to 100% because of rounding.

Table S3. Detailed results of positive rates by age group and subtype/lineage, Yichang, Hubei, 2013-2019.

Age group（years）, n(%)*	A/H1N1	A/H3N2	Victoria	Yamagata	Number of ILI patients tested
0-4	55(3.3)	85(5)	20(1.2)	29(1.7)	1690
5-9	81(7.7)	78(7.4)	46(4.4)	55(5.2)	1049
10-14	29(6.3)	33(7.1)	15(3.2)	29(6.3)	464
15-19	36(4.4)	35(4.3)	13(1.6)	26(3.2)	817
20-24	21(2.9)	34(4.7)	8(1.1)	20(2.8)	727
25-29	46(5)	51(5.6)	24(2.6)	6(0.7)	915
30-34	37(4.9)	41(5.5)	25(3.3)	14(1.9)	751
35-39	30(6.6)	15(3.3)	12(2.6)	7(1.5)	456
40-44	21(6.1)	18(5.2)	0(0)	12(3.5)	344
45-49	25(7.8)	22(6.9)	1(0.3)	5(1.6)	319
50-54	16(5.1)	19(6)	2(0.6)	12(3.8)	315
55-59	12(4.5)	15(5.7)	1(0.4)	13(4.9)	265
60-64	15(6)	20(8)	1(0.4)	13(5.2)	251
65-69	5(3.1)	13(8)	0(0)	8(4.9)	163
70-74	2(2.4)	6(7.1)	0(0)	5(5.9)	85
75-79	1(2.1)	2(4.3)	0(0)	1(2.1)	47
80-84	0(0)	3(10.7)	0(0)	0(0)	28
85+	0(0)	0(0)	0(0)	0(0)	7

*, Some columns do not add up to 100% because of rounding.

Table S4. Age distribution of influenza-positive patients in different surveillance years, Yichang, Hubei, 2013-2019.

Age group (years)[*], n (%)	2013-2014	2014-2015	2015-2016	2016-2017	2017-2018	2018-2019	Total
0-4	12(12.4)	28(19.6)	31(20.0)	18(12.9)	82(14.1)	33(10.2)	204(14.2)
5-14	47(48.5)	49(34.3)	56(36.1)	40(28.8)	137(23.5)	71(22.0)	400(27.8)
15-24	8(8.2)	11(7.7)	20(12.9)	20(14.4)	90(15.5)	53(16.4)	202(14.0)
25-59	25(25.8)	45(31.5)	45(29.0)	50(36.0)	213(36.6)	157(48.6)	535(37.2)
60+	5(5.2)	10(7.0)	3(1.9)	11(7.9)	60(10.3)	9(2.8)	98(6.8)

[*], Some columns do not add up to 100% because of rounding.

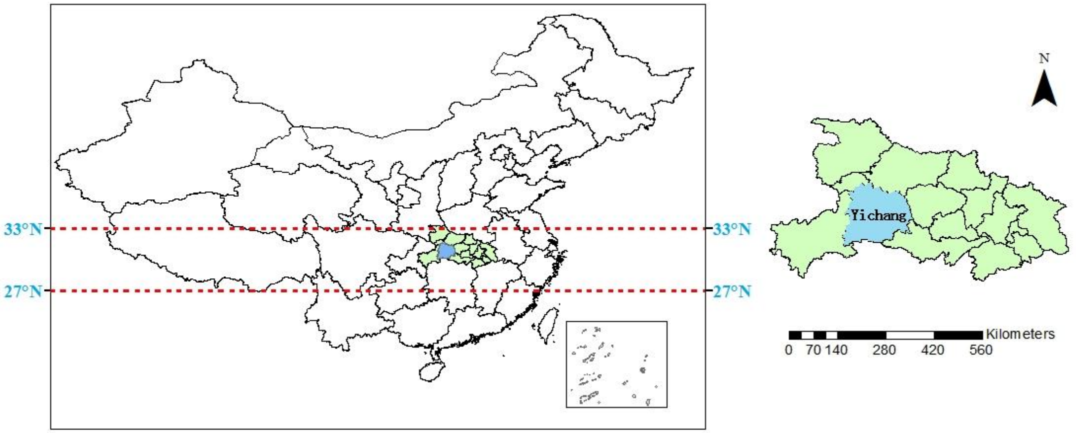

Figure S1. Location of Yichang city, Hubei Province, China.

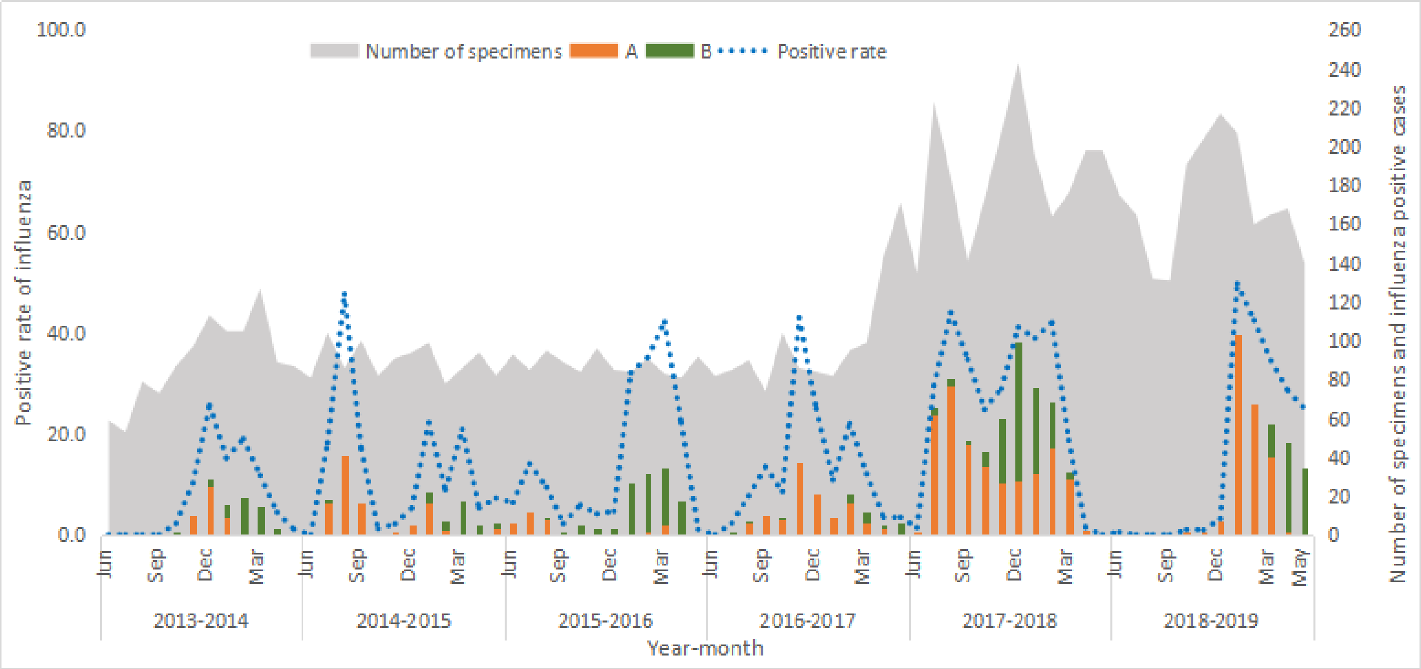

Figure S2. Temporal trends of influenza virus activity by type, Yichang, Hubei, 2013-2019. Influenza virus-positive cases by virus type (influenza viruses A and B). The shaded area represents the total number of specimens tested. The dark blue dotted line indicates the positive rate of influenza.

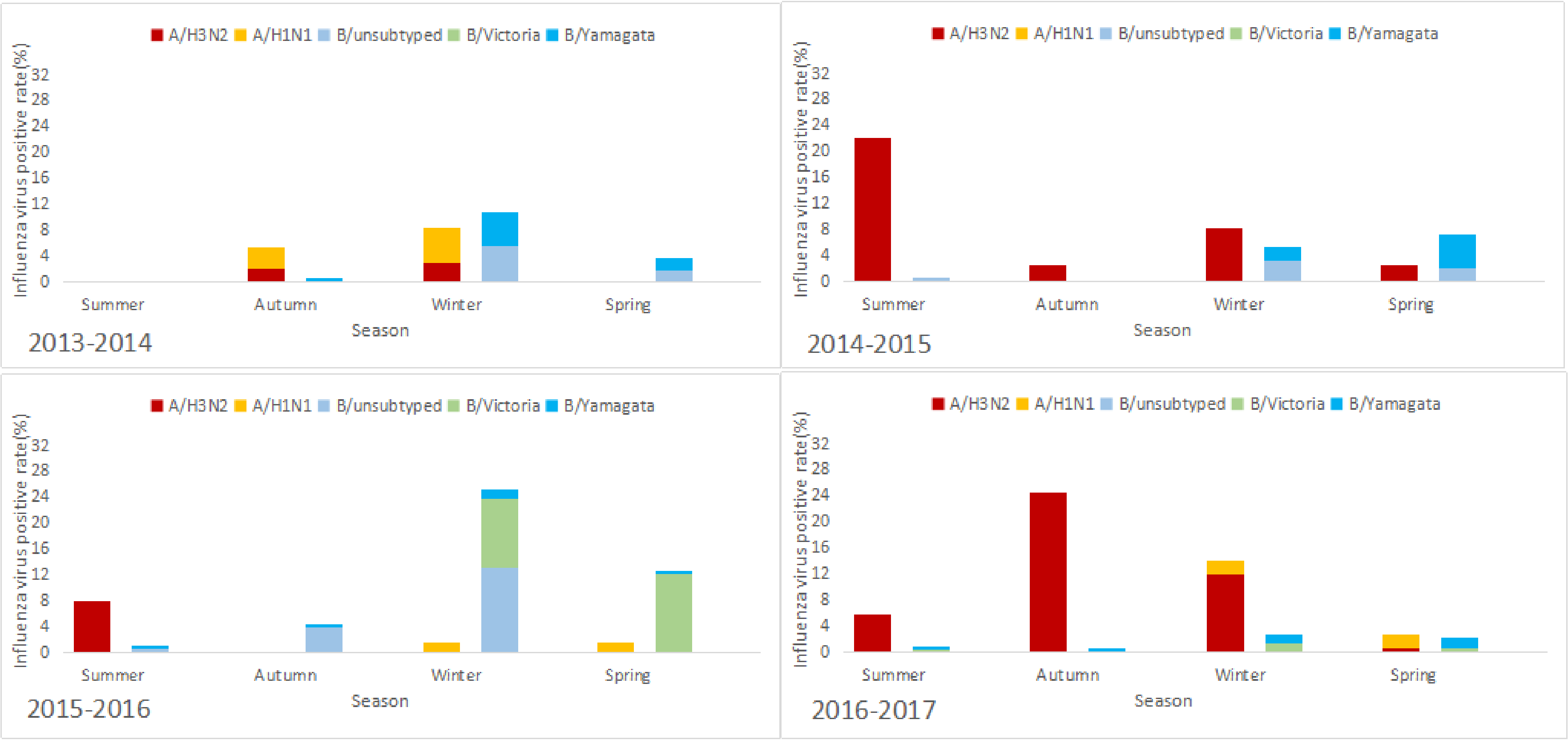
A/H3N2 A/H1N1 B/unsubtyped B/Victoria B/Yamagata
Influenza virus positive rate (%)
0 4 8 12 16 20 24 28 32
Summer Autumn Winter Spring
Season
2013-2014
2014-2015
2015-2016
2016-2017

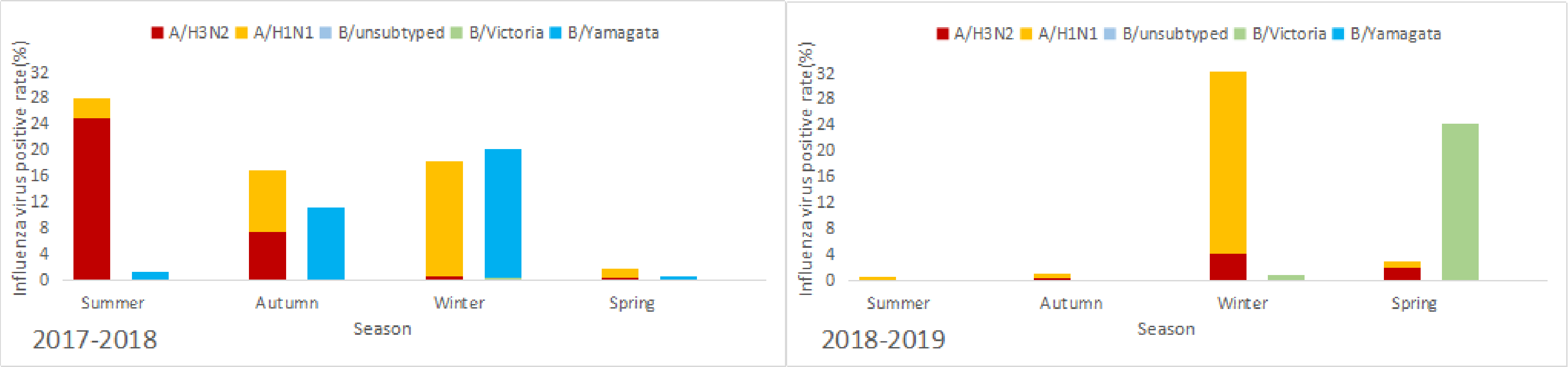

Figure S3. Distribution of positive rates by subtypes/lineages of influenza viruses A and B in different seasons in the six-year study period.

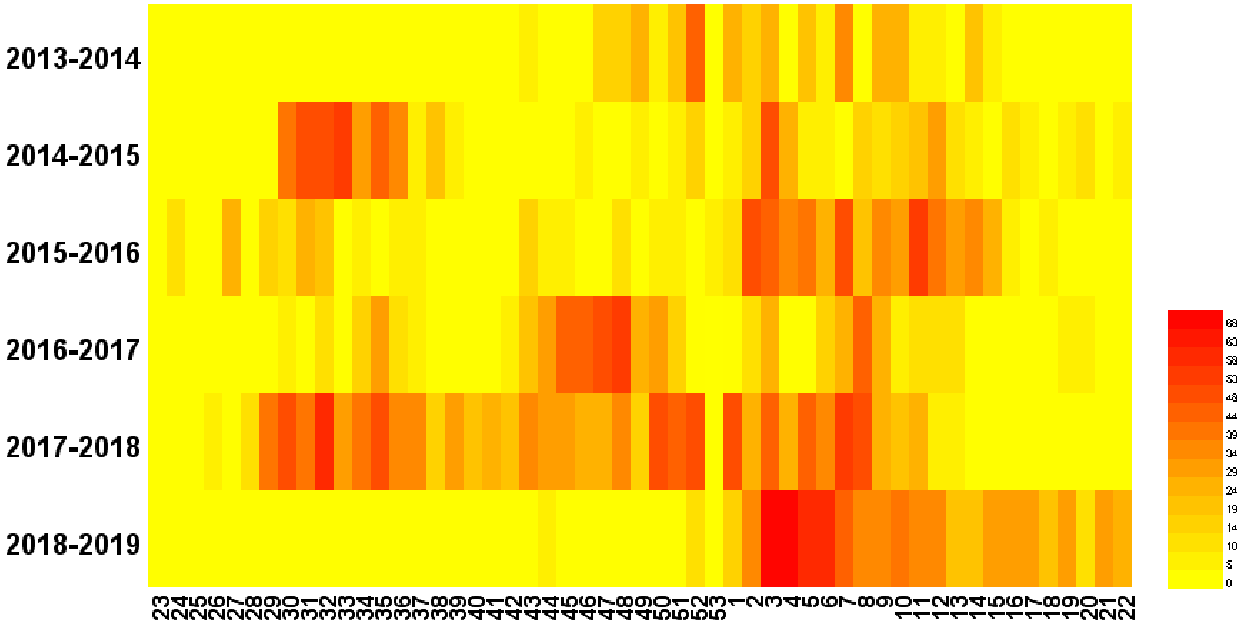

Figure S4. Heat map of influenza virus activity by week in Yichang, Hubei, 2013-2019.
